# Development and Field Validation of a Double‐Antigen Sandwich Colloidal Gold Immunochromatographic Strip for Detection of *Toxoplasma gondii* Antibodies in Multiple Host Species

**DOI:** 10.1155/tbed/5879710

**Published:** 2026-06-02

**Authors:** Xin Mu, Chen Chen, Xianglin Pu, Lixin Xu, Mingmin Lu, Ruofeng Yan, Xiangrui Li, Xiaokai Song

**Affiliations:** ^1^ MOE Joint International Research Laboratory of Animal Health and Food Safety, College of Veterinary Medicine, Nanjing Agricultural University, Nanjing, 210095, China, njau.edu.cn

**Keywords:** colloidal gold immunochromatographic assay (GICA), double-antigen sandwich, multispecies detection, point-of-care testing, *Toxoplasma gondii*

## Abstract

Toxoplasmosis, a globally prevalent zoonosis caused by *Toxoplasma gondii* (*T. gondii*), poses major threats to both human and animal health, leading to reproductive losses in livestock and severe disease in immunocompromised individuals. Although enzyme‐linked immunosorbent assay (ELISA) and PCR are widely used for diagnosis and surveillance, they may be limited by turnaround time, laboratory instrumentation, and, in the case of serological assays, the need for species‐specific reagents. To enable rapid, equipment‐minimal detection applicable to multiple host species, we developed a point‐of‐care (POC) double‐antigen sandwich colloidal gold immunochromatographic assay (GICA) based on recombinant surface antigen 2 (rSAG2) of *T. gondii*. In this assay, rSAG2 served both as the capture antigen immobilized on the test line and as the colloidal gold‐conjugated detection probe. Key parameters, including conjugation pH, antigen loading, and buffer composition, were systematically optimized. The resulting strip showed a detection limit of a 1:40 serum dilution, and no cross‐reactivity was observed with sera positive for 23 common pathogens from different host species. Good repeatability and storage stability for 4 months at 4°C were also observed. For field evaluation, 409 clinical serum samples from six animal groups (100 chickens, 68 dogs, 30 cats, 81 pigs, 80 yaks, and 50 sheep) were tested, and seropositivity rates ranged from 2.9% to 31.3% across the sampled groups. In a subset of chicken (*n* = 46) and dog (*n* = 44) sera tested in parallel with the corresponding commercial ELISA kits, the rSAG2‐GICA showed good preliminary agreement, with overall agreement rates of 91.3% and 97.7%, respectively. Collectively, this rSAG2‐based GICA shows potential as a rapid and practical tool for on‐site serological screening of *T. gondii* antibodies across multiple host species and may support epidemiological surveillance in diverse animal populations.

## 1. Introduction

Toxoplasmosis, caused by the protozoan parasite *Toxoplasma gondii* (*T. gondii*), is a globally distributed zoonosis of major public health significance and substantial veterinary economic burden. Ranked as the fourth most important food‐borne parasite in a joint FAO/WHO report [1], *T. gondii* is estimated to infect approximately one‐third of the world’s human population [2]. Although infection in immunocompetent individuals is often mild or asymptomatic, it can be life‐threatening in vulnerable groups. In immunocompromised patients, reactivation of latent infection may result in severe disease, including toxoplasmic encephalitis, whereas infection during pregnancy can lead to abortion or congenital abnormalities [3, 4]. In livestock, *T. gondii* is an important cause of reproductive failure, manifesting as abortion, fetal death, and congenital malformations, and thereby contributes to substantial economic losses [5]. Transmission occurs primarily through ingestion of tissue cysts in undercooked meat from food‐producing animals (e.g., poultry, pigs, cattle, and sheep) or through ingestion of oocysts shed in the feces of definitive feline hosts that contaminate the environment, food, and water [6]. Accordingly, effective surveillance and control of toxoplasmosis depend on accessible and reliable diagnostic tools.

Clinical diagnosis remains challenging because toxoplasmosis lacks pathognomonic symptoms [7]. Current laboratory approaches include direct detection, molecular methods, and serology. Direct microscopic examination is insensitive and subjective, limiting its routine applicability [8]. The mouse or cat inoculation bioassay, historically regarded as a reference method, is time‐consuming, costly, and constrained by ethical considerations, which precludes large‐scale use [9]. Serological assays, particularly enzyme‐linked immunosorbent assay (ELISA), are therefore widely adopted for screening and surveillance. However, conventional ELISAs often rely on tachyzoite lysate antigens, which may compromise accuracy due to host‐cell contamination or antigenic cross‐reactivity [10, 11]. Moreover, many serological assays require species‐specific reagents, complicating multispecies surveillance. Although PCR‐based methods offer high analytical specificity, they require specialized equipment and technical expertise, limiting their deployment in field or point‐of‐care (POC) settings.

Colloidal gold immunochromatographic assays (GICAs) have emerged as rapid, instrument‐free platforms well suited to POC testing. Compared with ELISA, GICA strips can yield results within minutes, are generally stable at ambient temperature, reduce per‐test cost, and enable visual interpretation without dedicated instrumentation [12, 13]. These attributes make GICA attractive for resource‐limited settings and large‐scale screening [14, 15]. Importantly, GICA performance depends heavily on antigen selection. The surface antigen 2 (SAG2) of *T. gondii* is a well‐characterized, immunodominant target with high conservation, strong immunogenicity, and expressed during infection [16, 17]. SAG2 has been used successfully in multiple immunoassays and has demonstrated high sensitivity and specificity with minimal cross‐reactivity across species, supporting its suitability for broad‐spectrum serodiagnosis [18, 19].

To address the need for a rapid and species‐independent POC test for toxoplasmosis, we developed a double‐antigen sandwich GICA strip based on recombinant SAG2 (rSAG2). In this format, rSAG2 was used both as the capture antigen on the test line and, after conjugation with colloidal gold nanoparticles, as the detection probe, thereby enabling cross‐species detection of anti‐*T. gondii* antibodies without the need for species‐specific secondary reagents. After systematic optimization of key parameters, the strip was evaluated for detection limit, analytical specificity against the tested serum panel, repeatability, and stability. Its performance was further assessed using field serum samples from six animal groups, including chickens, dogs, cats, pigs, yaks, and sheep, and preliminary comparator‐based agreement was examined in subsets of chicken and dog sera using the corresponding commercial ELISA kits. These findings suggest that the developed assay has potential as a practical tool for rapid serological screening and epidemiological surveillance of *T. gondii* infection in diverse animal hosts.

## 2. Materials and Methods

### 2.1. Culture and Isolation of *T. gondii* Tachyzoites

Human foreskin fibroblast (HFF) cells (Stem Cell Bank, Chinese Academy of Sciences, China) were maintained in Dulbecco’s Modified Eagle Medium (DMEM; Thermo Fisher Scientific, MA, United States) supplemented with 10% fetal bovine serum (FBS; Thermo Fisher Scientific, MA, United States) and 1% penicillin–streptomycin at 37°C in a humidified incubator with 5% CO_2_. When the HFF monolayers reached 70%–80% confluence, the culture medium was removed, and the cells were gently washed twice with 3 mL of pre‐warmed (37°C) phosphate‐buffered saline (PBS) to remove residual serum. Tachyzoites of the *T. gondii* RH strain (kindly provided by Prof. Bang Shen, Huazhong Agricultural University, Wuhan, China) were then inoculated onto the HFF monolayers, and a complete medium was added to a final volume of 12 mL. The infected cells were returned to the incubator and examined daily under a microscope for host cell lysis and tachyzoite egress. Fresh complete medium was added when necessary to maintain the cultures.

After 5–6 days of incubation, when extensive intracellular proliferation and parasite egress were observed, the cultures were harvested. The cell monolayers were detached using a sterile cell scraper, and the resulting suspensions were transferred into sterile 50 mL centrifuge tubes. The suspensions were centrifuged at 3900 r/min for 8 min at 4°C, and the pellets were resuspended in 10 mL of sterile PBS. To release the remaining intracellular tachyzoites and disrupt residual host cells, the suspensions were passed 30 times through a sterile 27‐gauge needle. The resulting suspension was then filtered through a 5 μm membrane to remove host cell debris and larger impurities. The filtrate was collected and centrifuged again at 3900 r/min for 8 min at 4°C. Finally, the pellet was resuspended in 5 mL of sterile PBS to obtain purified *T. gondii* RH tachyzoites for subsequent experiments.

### 2.2. Preparation and Antibody Titer Determination of *T. gondii*‐Infected Sera

To generate *T. gondii*‐specific antisera, rats and chickens were experimentally infected with tachyzoites of the RH strain. Rats were intraperitoneally injected with 2 × 10^6^ tachyzoites, whereas chickens were intraperitoneally injected with 1 × 10^8^ tachyzoites. At 21 days post‐infection, blood was collected from rats via the abdominal aorta and from chickens via cardiac puncture. The samples were allowed to clot at 37°C for 30 min, followed by standing overnight at 4°C. After serum separation, the serum was carefully harvested, aliquoted, and stored at −80°C until further use.

Antibody titers of *T. gondii*‐infected rat and chicken sera were determined by indirect ELISA. Briefly, ELISA plates were coated overnight at 4°C with 100 μL per well of rSAG2 diluted to 4 μg/mL in 0.05 mol/L carbonate buffer. After washing with PBST (PBS containing 0.5% Tween‐20), the plates were blocked with 5% skim milk at 37°C for 1 h. The infected and negative sera were then serially diluted and added to the plates, followed by incubation at 37°C for 1 h. After washing, HRP‐conjugated goat anti‐rat IgG or rabbit anti‐chicken IgY secondary antibody (1:5000; Proteintech, Wuhan, China) was added and incubated at 37°C for 1 h. Following further washes, TMB substrate was added for color development in the dark for 15 min, and the reaction was terminated with stop solution. OD_450_ was measured using a microplate reader, and the antibody titer was defined as the highest dilution at which the test serum OD_450_ exceeded 2.1 times that of the negative control at the same dilution.

### 2.3. Construction of the SAG2 Expression Plasmid

Total RNA was extracted from purified *T. gondii* RH strain tachyzoites using the TRIzol reagent (Vazyme, Nanjing, China) and quantified using a NanoDrop spectrophotometer. First‐strand cDNA was synthesized from 5 μg total RNA using an Oligo(dT)18 primer and a commercial reverse transcription kit (Thermo Fisher Scientific, MA, United States) according to the manufacturer’s instructions. The coding sequence of the SAG2 gene (GenBank: FJ825705), excluding the signal peptide, was amplified from cDNA by PCR using PrimeSTAR Max DNA polymerase (TaKaRa, Dalian, China). Primers were designed to include SacI and HindIII restriction sites at their 5^′^ ends and are listed in Supporting Information 1: Table S1. PCR was performed under the following conditions: initial denaturation at 98°C for 3 min; 30 cycles of 98°C for 10 s, 55°C for 5 s, and 72°C for 35 s; and a final extension at 72°C for 5 min. The amplified SAG2 fragment and the pET‐32a(+) vector (Invitrogen, CA, United States) were digested with SacI and HindIII (TaKaRa, Dalian, China), gel‐purified, and ligated using T4 DNA ligase (TaKaRa, Dalian, China) to generate the recombinant plasmid pET‐32a‐SAG2. The ligation mixture was transformed into competent *E. coli* BL21 (DE3) cells (Invitrogen, CA, United States), and transformants were selected on LB agar plates containing 100 μg/mL ampicillin. Recombinant clones were initially screened by SacI and HindIII digestion to confirm insert release, and correct insertion and sequence integrity were further verified by bidirectional Sanger sequencing.

### 2.4. Expression, Purification, and Immunoreactivity Identification of rSAG2

*E. coli* BL21 (DE3) cells harboring pET‐32a‐SAG2 were cultured in a LB medium supplemented with ampicillin (100 μg/mL) at 37°C with shaking at 180 rpm until the OD_600_ reached 0.6–0.8. rSAG2 expression was induced by adding isopropyl β‐D‐thiogalactoside (IPTG; Yfxbio Biotech Co., Ltd., Nanjing, China) to a final concentration of 1 mM. Large‐scale expression was subsequently performed under the same induction conditions. After 5 h of induction, the cells were harvested and lysed by ultrasonication. rSAG2 was purified using a Ni‐NTA affinity column (Cytiva, MA, USA), and purity was assessed by 12% SDS–PAGE.

Immunoreactivity of purified rSAG2 was confirmed by western blot. Proteins were resolved by 12% SDS–PAGE and transferred onto a PVDF membrane (Merck, Darmstadt, Germany) using a semi‐dry transfer system (Trans‐Blot Turbo, Bio‐Rad, Hercules, CA, USA). Membranes were blocked with 5% (w/v) skim milk (Solarbio, Beijing, China) for 2 h at room temperature and incubated overnight at 4°C with *T. gondii*‐positive rat serum (1:100), *T. gondii*‐positive chicken serum (1:100), or monoclonal anti‐His‐tag antibody (1:5000). Negative rat and chicken sera served as controls. After washing five times with TBST (TBS buffer containing 0.5% Tween‐20), membranes were incubated with the corresponding HRP‐conjugated secondary antibodies (Proteintech, Wuhan, China): goat anti‐rat IgG (1:10,000), goat anti‐chicken IgY (1:10,000), or goat anti‐mouse IgG (1:10,000). Signals were developed using Chemistar High‐sig ECL western blotting substrate (Tanon, Shanghai, China) and imaged with a Tanon 5200 series chemiluminescence imaging system (Tanon, Shanghai, China).

### 2.5. Preparation, Purification, and Validation of Rabbit Anti‐rSAG2 Polyclonal IgG

Rabbit anti‐rSAG2 polyclonal antibodies were generated in two male New Zealand White rabbits. Following a 2‐week acclimatization period, pre‐immune serum was collected as a negative control. Rabbits were subcutaneously immunized with 500 μg purified rSAG2 emulsified in Freund’s complete adjuvant (Merck, Darmstadt, Germany), followed by three booster immunizations at 2‐week intervals using 250 μg antigen emulsified in Freund’s incomplete adjuvant. Terminal blood collection was performed 10 days after the final booster, and sera were separated, aliquoted, and stored at −80°C. Antibody titers were determined by indirect ELISA.

Rabbit IgG was purified by caprylic acid‐ammonium sulfate precipitation (CAAS). Briefly, anti‐rSAG2 antiserum was thawed and mixed with three volumes of pre‐chilled 0.06 M sodium acetate buffer (pH 4.8). The pH was adjusted to 4.5, and caprylic acid was added dropwise to a final concentration of 2.5% (v/v) under continuous stirring for 30 min. After incubation at 4°C for 2 h, the mixture was centrifuged at 8000 × *g* for 30 min. The supernatant was filtered through a 0.22 μm membrane and diluted 10‐fold with 0.01 M PBS (pH 7.4), followed by readjustment of pH to 7.4. Saturated ammonium sulfate solution was then added dropwise under constant stirring to precipitate IgG, and the mixture was incubated overnight at 4°C. After centrifugation at 12,000 × *g* for 15 min, the pellet was dissolved in 0.01 M phosphate buffer (pH 7.4) and dialyzed extensively against the same buffer using a 3.5 kDa cutoff membrane. Purified IgG was filter‐sterilized, quantified by the BCA assay, assessed by SDS–PAGE, and stored at −80°C.

The reactivity of the purified rabbit IgG was confirmed by western blot using rSAG2 as the antigen. After SDS–PAGE and transfer onto a PVDF membrane, the membrane was incubated overnight at 4°C with purified rabbit IgG (1:100), followed by incubation with HRP‐conjugated goat anti‐rabbit IgG (1:10,000; Proteintech, Wuhan, China) for 1 h at room temperature. After TBST washes, signals were developed with the ECL substrate and imaged using a chemiluminescence imaging system.

### 2.6. Optimization of pH and Antigen Amount for Colloidal Gold Labeling

The optimal pH for colloidal gold labeling was determined by adjusting 125 μL aliquots of 20 nm colloidal gold solution to pH 4.0–9.0 with 0.1 M HCl or K_2_CO_3_, followed by incubation with 10 μg rSAG2 for 20 min at room temperature. Afterward, 125 μL of 10% (w/v) NaCl was added to each mixture to give a final NaCl concentration of 5%, and the samples were incubated in the dark for 2 h. Colloidal stability was assessed by visual observation of color change and by measuring absorbance at 520 nm (OD_520_). The optimal pH was defined as the lowest pH at which salt‐induced aggregation was prevented, as indicated by the retention of the original burgundy color and a high OD_520_ value.

The optimal antigen labeling amount was then determined using colloidal gold pre‐adjusted to the optimal pH with 0.1 M K_2_CO_3_. Briefly, 125 μL aliquots of colloidal gold were incubated with increasing amounts of rSAG2 (0–4.5 μL of a 1600 μg/mL solution) for 20 min at room temperature. Subsequently, 125 μL of 10% (w/v) NaCl was added to each mixture to a final concentration of 5%, followed by incubation at room temperature for 2 h. Colloidal stability was evaluated by visual inspection and OD_520_ measurements. The optimal antigen amount was defined as the minimum quantity of rSAG2 required to prevent salt‐induced aggregation, as indicated by the maintenance of a stable burgundy color and a high OD_520_ value.

### 2.7. Preparation of Gold‐Labeled Antigen

A 1 mL aliquot of colloidal gold solution was adjusted to the predetermined optimal pH using 0.1 M K_2_CO_3_ and equilibrated for 5 min. rSAG2 was then added (14.4 μL of the protein solution) and incubated for 30 min at room temperature to allow conjugation. Subsequently, 100 μL of 10% (w/v) BSA was added dropwise to block the unoccupied binding sites on the gold nanoparticles. After an additional 30 min, the mixture was centrifuged at 12,000 × *g* for 30 min at 4°C. The supernatant was carefully discarded, and the pellet was resuspended in 100 μL of resuspension buffer. The gold–rSAG2 conjugate was stored at 4°C until use.

### 2.8. Preparation of rSAG2–GICA Strips

Glass fiber pads (Jieyi Biotechnology Co., Ltd., Shanghai, China) were used as the sample pad and conjugate pads. The sample pad was pretreated with buffer (0.01 M phosphate buffer containing 1% BSA and 1% Tween‐20, pH 7.4), and the conjugate pad was pretreated with the designated processing buffer. Both pads were dried at 37°C for 3 h. The conjugate pad was then coated with the gold‐labeled antigen (200 μL per 5 cm pad) and dried again at 37°C for 3 h. All processed pads were sealed in aluminum foil pouches containing desiccant and stored at 4°C until assembly.

The nitrocellulose (NC) membrane (Jieyi Biotechnology Co., Ltd., Shanghai, China) was laminated onto a PVC backing card and equilibrated at room temperature for 1 h. Using an XYZ dispensing system (HM3260, Kinbio Tech Co., Ltd., Shanghai, China), purified rabbit anti‐rSAG2 IgG was dispensed onto the control (C) line, and rSAG2 was dispensed onto the test (T) line. The coated membrane was dried at 37°C for 3 h and stored at room temperature in the dark. The sample pad, conjugate pad, NC membrane, and absorbent pad were assembled on the backing card with appropriate overlaps. The assembled cards were cut into 4.0‐mm‐wide strips using a microcomputer‐controlled automatic cutter (ZQ2002, Kinbio Tech Co., Ltd., Shanghai, China). Individual strips were mounted into plastic cassettes, sealed in aluminum foil pouches with desiccant, and stored for future use.

### 2.9. Optimization of rSAG2–GICA Strip Working Conditions

The conjugate pad pretreatment buffer was optimized by evaluating nine formulations containing different concentrations of sucrose, BSA, and Tween‐20. Formulations were selected based on signal intensity with positive samples and background with negative samples. The resuspension buffer for the gold‐labeled antigen was similarly selected from three candidate formulations according to the overall color development performance. The optimal resuspension volume was determined by resuspending the gold‐labeled antigen (prepared from 1 mL of colloidal gold solution) in different volumes (300, 400, 600, 800, and 1000 μL). After coating the conjugate pads and assembling the strips, performance was compared using positive and negative samples to identify the optimal resuspension volume.

The optimal coating concentrations for the test (T) and control (C) lines were determined by dispensing concentration gradients onto the NC membrane. The T line was coated with rSAG2 at 300, 600, 900, 1300, and 1500 μg/mL, while the C line was coated with rabbit anti‐rSAG2 IgG at 2, 3, 4, 5, and 6 mg/mL. After drying and strip assembly, each condition was evaluated by testing positive and negative samples and comparing the line intensity and nonspecific background.

Finally, the sample dilution/running buffer and its pH were optimized sequentially. Five candidate buffers (PB, PBS, PBST, TBST, and Tris‐HCl) were evaluated by testing positive and negative sera diluted in each buffer (in triplicate). The optimal buffer was selected based on the chromatographic flow performance and line intensity. The pH of the selected buffer was then optimized by adjusting it to 3.0, 5.0, 7.0, 9.0, and 11.0 using 0.1 M HCl or KOH. Positive and negative sera were diluted using each pH‐adjusted buffer and tested in triplicate, and the optimal pH was determined based on the test‐line intensity while minimizing background.

### 2.10. Detection Limit and Specificity of the rSAG2–GICA Strips

To evaluate the detection limit, *T. gondii*‐infected rat serum was serially diluted (1:2, 1:4, 1:8, 1:16, 1:32, 1:40, and 1:64) in the sample dilution buffer and tested using the rSAG2–GICA strips, with all reactions performed in triplicate. The detection limit was defined as the highest dilution at which a visible test (T) line was observed, with a valid control (C) line present.

Specificity was assessed using strips from the same production batch by testing sera positive for the following 23 animal pathogens: *Eimeria tenella* (*E. tenella*), *Eimeria necatrix* (*E. necatrix*), *Eimeria maxima* (*E. maxima*), *Eimeria acervulina* (*E. acervulina*), infectious bursal disease virus (IBDV), Newcastle disease virus (NDV), *Salmonella* spp., *Pasteurella multocida* (*P. multocida*), *Trichinella spiralis* (*T. spiralis*), *Haemonchus contortus* (*H. contortus*), *Actinobacillus pleuropneumoniae* (*A. pleuropneumoniae*), classical swine fever virus (CSFV), pseudorabies virus (PRV), porcine reproductive and respiratory syndrome virus (PRRSV), porcine circovirus (PCV), *Streptococcus suis* (*S. suis*), *Glaesserella parasuis* (*G. parasuis*), canine parvovirus (CPV), canine adenovirus (CADV), canine influenza virus (CIV), feline calicivirus (FCV), feline parvovirus (FPV), and feline herpesvirus (FHV). Among them, sera positive for *Eimeria* spp., IBDV, NDV, and *Salmonella* spp. were derived from chickens; sera positive for *P. multocida* from rabbits; sera positive for *T. spiralis* from rats; sera positive for *H. contortus* from sheep; sera positive for *A. pleuropneumoniae*, CSFV, PRV, PRRSV, PCV, *S. suis*, and *G. parasuis* from pigs; sera positive for CPV, CADV, and CIV from dogs; and sera positive for FCV, FPV, and FHV from cats. In addition, all sera were diluted with TBST at a volume ratio of 1:7.5 before testing, and all reactions were performed in duplicate. Any visible T‐line signal produced by these sera was recorded as cross‐reactivity. *T. gondii*‐positive and negative sera were included as controls in all specificity assays.

### 2.11. Repeatability and Stability of the rSAG2–GICA Strips

The repeatability and stability of the rSAG2–GICA strips were evaluated. Repeatability was assessed at both the intra‐assay and inter‐assay levels. For intra‐assay repeatability, multiple strips from the same production batch were tested using *T. gondii*‐positive and negative serum samples. For inter‐assay repeatability, strips from three independent production batches were tested using the same serum panel. For stability evaluation, strips were sealed in foil pouches containing desiccant and stored at 4°C. Performance was assessed monthly by testing stored strips with positive and negative control sera.

### 2.12. Detection of Clinical Serum Samples

To assess clinical performance, the optimized rSAG2–GICA strips were used to detect *T. gondii* antibodies in sera collected from multiple animal species. Before testing, serum samples were diluted with TBST at a volume ratio of 1:7.5. The assay was performed at room temperature (25°C), and the results were recorded within 10–15 min. The serum panel included 100 chickens (Nantong, Jiangsu Province), 80 yaks (Sichuan Province), 81 pigs (Jiangsu Province), 50 sheep (Shandong Province), 68 dogs (Suzhou, Jiangsu Province), and 30 cats (Nanjing, Jiangsu Province). Seropositivity was further analyzed according to animal species, husbandry practices, and geographical regions.

### 2.13. Comparative Detection Using Commercial ELISA Kits

A subset of the chicken (*n* = 46) and dog (*n* = 44) serum samples was tested in parallel using commercial ELISA kits (Chicken Tox‐Ab ELISA Kit and Canine Tox‐Ab ELISA Kit; Shanghai Enzyme‐linked Biotechnology Co., Ltd., Shanghai, China) according to the manufacturer’s instructions, and the results were compared with those obtained by the rSAG2–GICA strips for preliminary method comparison.

### 2.14. Statistical Analysis

For the clinical serum samples, seropositivity was calculated as the proportion of positive samples among the total number tested in each subgroup. The 95% confidence intervals (95% CIs) were calculated using the exact Clopper–Pearson binomial method. Differences in seropositivity among animal species were evaluated using Pearson’s chi‐square test. Because host species, breeding system, and sampling region were not independent in the present sampling design, the results stratified by breeding system and region were interpreted mainly descriptively. In addition, the comparison between free‐range and intensively reared chickens was analyzed using Fisher’s exact test because of the relatively small sample size in the free‐range chicken subgroup.

For the subset of chicken and dog serum samples tested in parallel by rSAG2–GICA and the corresponding commercial ELISA kits, diagnostic sensitivity, diagnostic specificity, and overall agreement were calculated using the commercial ELISA as the reference comparator. The diagnostic sensitivity was calculated as the proportion of ELISA‐positive samples that were also positive by rSAG2–GICA. Diagnostic specificity was calculated as the proportion of ELISA‐negative samples that were also negative by rSAG2–GICA. The overall agreement was calculated as the proportion of samples with concordant results between rSAG2–GICA and commercial ELISA among all tested samples. The 95% CI for overall agreement was calculated using the exact Clopper–Pearson binomial method. A paired comparison between rSAG2–GICA and commercial ELISA was performed using McNemar’s exact test. A two‐sided *p*  < 0.05 was considered statistically significant.

## 3. Results

### 3.1. Antibody Titers of *T. gondii*‐Infected Chicken and Rat Sera Reached 1:6400

To determine the antibody titers of the experimentally prepared sera from *T. gondii*‐infected chickens and rats, an indirect ELISA was performed. The results showed that both the infected rat serum and the infected chicken serum remained positive at a dilution of 1:6400, indicating that the prepared positive sera had sufficiently high antibody titers for subsequent assays.

### 3.2. SAG2 Was Successfully Cloned Into the pET‐32a Expression Plasmid

The SAG2 gene was successfully amplified by PCR, yielding a single product of approximately 480 bp (Supporting Information 2: Figure S1A, lane 1). The recombinant plasmid pET‐32a‐SAG2 was verified by double restriction enzyme digestion, which released the expected 480 bp insert (Supporting Information 2: Figure S1A, lane 2). Sequence analysis further confirmed correct insertion, showing 100% identity to the reference SAG2 sequence (GenBank: FJ825705).

### 3.3. rSAG2 Was Successfully Expressed, Purified, and Demonstrated Good Antigenicity

**Figure 1:**
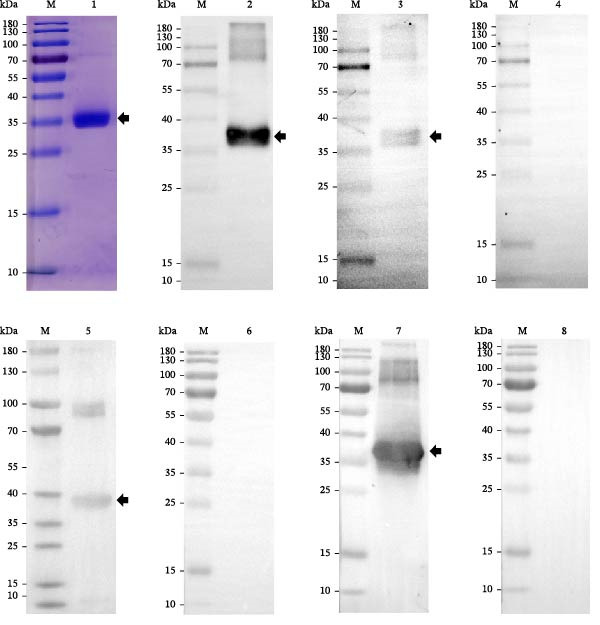
Expression, purification, and immunoreactivity identification of rSAG2. M: protein molecular weight marker. Lane 1: purified recombinant rSAG2 (SDS–PAGE). Lane 2: western blot detection of rSAG2 using an anti‐His‐tag antibody. Lane 3: western blot detection of rSAG2 using *T. gondii*‐infected rat serum. Lane 4: western blot detection using naïve rat serum. Lane 5: western blot detection of rSAG2 using *T. gondii*‐infected chicken serum. Lane 6: western blot detection using naïve chicken serum. Lane 7: western blot detection of rSAG2 using purified rabbit anti‐rSAG2 polyclonal IgG. Lane 8: western blot detection using naïve rabbit serum.

rSAG2 was successfully expressed in *E. coli* BL21 (DE3) and purified by Ni‐NTA affinity chromatography. SDS–PAGE analysis showed that rSAG2 was distributed in both the soluble fraction and inclusion bodies after induction (Supporting Information 2: Figure S1B), and purification from the soluble fraction yielded a predominant band at the expected theoretical molecular weight of the target protein (Figure 1, lane 1). The identity and immunoreactivity of rSAG2 were further confirmed by western blot analysis. As shown in Figure 1, immunoreactive bands close to the expected molecular weight range of rSAG2 were detected by the anti‐His‐tag antibody (lane 2) and were also recognized by *T. gondii*‐positive rat serum, *T. gondii*‐positive chicken serum, and purified rabbit anti‐rSAG2 polyclonal IgG (lanes 3, 5, and 7, respectively), whereas no corresponding band was observed with the respective negative‐control sera (lanes 4, 6, and 8). In lane 5, two reactive bands were observed. The lower band was considered more likely to correspond to rSAG2, although its apparent molecular weight was slightly higher than the theoretical value, which may reflect a minor difference between the theoretical and apparent molecular weights during SDS–PAGE and western blot analysis. The upper band was an additional immunoreactive band, the identity of which could not be conclusively determined in the present study, and it may represent nonspecific reactivity or a higher‐molecular‐weight form of the recombinant protein. In addition, indirect ELISA showed that the titer of rabbit anti‐rSAG2 polyclonal antibodies reached 1:12,800. These results indicated that rSAG2 was successfully expressed and purified and could be specifically recognized by different positive sera and purified rabbit polyclonal antibody.

### 3.4. The Optimal Working Conditions for the GICA Were Determined

As shown in Supporting Information 3: Figure S2A, the highest OD_520_ value (0.346) was obtained when 2 μL of 0.1 M K_2_CO_3_ was added to 125 μL of colloidal gold solution (equivalent to 16 μL/mL), and the conjugate remained stable after the addition of NaCl to a final concentration of 5%, indicating that the optimal labeling pH was 6.0 ± 0.2, which was close to the predicted isoelectric point of rSAG2 (pI = 5.63). As shown in Supporting Information 3: Figure S2B, the highest OD_520_ value (0.288) was obtained at an rSAG2 concentration of 19.2 μg/mL; however, for practical application, the labeling concentration was set at 23.04 μg/mL, representing a 20% increase over the minimum concentration required to prevent aggregation. Under these optimized conditions, the conjugate pad was pretreated with 0.01 M PB containing 3% sucrose, 5% BSA, and 5% Tween‐20, and the gold‐labeled antigen was resuspended in 0.01 M PB supplemented with 5% sucrose, 2% BSA, and 1% Tween‐20 at a volume of 600 μL per 1 mL of colloidal gold solution. In addition, the optimal coating concentrations were 0.9 mg/mL rSAG2 for the test line and 4 mg/mL rabbit anti‐rSAG2 IgG for the control line, with TBST used as the sample dilution buffer and pH 7.0 as the optimal running buffer condition.

### 3.5. The Developed GICA Showed a Detection Limit of a 1:40 Serum Dilution

**Figure 2:**
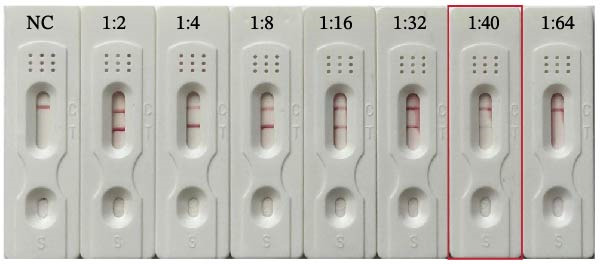
Detection limit of the rSAG2–GICA strips using serially diluted *T. gondii*‐positive serum. Representative photographs show the results of the rSAG2‐GICA strips tested with serial dilutions of *T. gondii*‐positive serum. NC, negative control. Strips 1–7 correspond to serum dilutions of 1:2, 1:4, 1:8, 1:16, 1:32, 1:40, and 1:64, respectively. The experiment was performed in triplicate. The detection limit was defined as the highest serum dilution that still produced a visible test (T) line in the presence of a valid control (C) line.

The detection limit was evaluated using serial dilutions of *T. gondii*‐infected rat serum. As shown in Figure 2, the test (T) line exhibited strong signals at dilutions up to 1:16. The signal intensity decreased progressively with increasing dilution, and a faint but discernible T line was still observed at 1:40. No visible T line was observed at a 1:64 dilution. Therefore, the detection limit of the strip was preliminary determined to be 1:40.

### 3.6. The Developed GICA Showed Good Specificity With no Detectable Cross‐Reactivity

Specificity was assessed using sera positive for 23 common animal pathogens derived from different host species. All sera were diluted with TBST at a volume ratio of 1:7.5 and tested under the same conditions. As shown in Supporting Information 4: Figure S3, no visible test (T) line was observed for any of the non‐*T. gondii*‐positive sera, while the *T. gondii*‐positive serum produced a clear positive result, and the negative serum showed only the control (C) line. These findings demonstrated that the developed rSAG2–GICA strip had good analytical specificity, with no detectable cross‐reactivity against sera positive for the other tested pathogens.

### 3.7. The Developed GICA Showed Good Repeatability and Stability

Repeatability was assessed at both the intra‐assay and inter‐assay levels. Strips from the same production batch generated consistent results when tested with *T. gondii*‐positive and negative sera. Similarly, strips from three independent production batches showed comparable performance with the same serum panel, demonstrating good batch‐to‐batch reproducibility.

Stability was evaluated by storing the strips at 4°C in sealed pouches with desiccant and testing them at regular intervals. The strips maintained a reliable performance after 4 months of storage, indicating good storage stability.

### 3.8. The Developed GICA Revealed Species‐Related Differences in *T. gondii* Seroprevalence

The optimized rSAG2–GICA strips were applied to 409 clinical serum samples collected from six animal species. As summarized in Table 1, the seropositivity rates were 7.0% (7/100; 95% CI: 2.9%–13.9%) in chickens, 25.9% (21/81; 95% CI: 16.8%–36.9%) in pigs, 2.9% (2/68; 95% CI: 0.4%–10.2%) in dogs, 3.3% (1/30; 95% CI: 0.1%–17.2%) in cats, 26.0% (13/50; 95% CI: 14.6%–40.3%) in sheep, and 31.3% (25/80; 95% CI: 21.3%–42.6%) in yaks. Overall, seropositivity differed significantly among animal species (Pearson’s chi‐square test, *χ* ^2^ = 39.78, df = 5, *p*  < 0.001). Descriptively, seropositivity was higher in free‐range animals, including free‐range chickens (40.0%, 4/10), sheep (26.0%, 13/50), and yaks (31.3%, 25/80), than in intensively reared chickens (3.3%, 3/90), while intensively reared pigs showed a seropositivity rate of 25.9% (21/81). In household‐reared companion animals, the seropositivity rates were relatively low, at 2.9% (2/68) in dogs and 3.3% (1/30) in cats. Geographically, positive samples were identified in all three provinces, with the detailed distribution shown in Table 1.

**Table 1:** Sample distribution and seropositivity of *Toxoplasma gondii* in clinical sera.

Region	Species	Breeding system	Sample amount	Positive	Prevalence (%)	95% CI (％)
Jiangsu	Chicken	Intensive farming	90	3	3.3	0.7–9.4
	Chicken	Free‐range	10	4	40.0	12.2–73.8
	Pig	Intensive farming	81	21	25.9	16.8–36.9
	Dog	Household rearing	68	2	2.9	0.4–10.2
	Cat	Household rearing	30	1	3.3	0.1–17.2
Shandong	Sheep	Free‐range	50	13	26.0	14.6–40.3
Sichuan	Yak	Free‐range	80	25	31.3	21.3–42.6

*Note:* Seropositivity was determined using the optimized rSAG2–GICA strips. Prevalence was calculated as the number of positive samples divided by the total number of samples tested in each subgroup. The 95% confidence intervals (95% CIs) were calculated using the exact Clopper–Pearson binomial method. Because host species, breeding system, and sampling region were not independent in the present study design, the results by breeding system and region are presented descriptively. Overall differences in seropositivity among animal species were evaluated separately using Pearson’s chi‐square test.

### 3.9. The Developed GICA Showed Good Preliminary Agreement With Commercial ELISA Kits

Comparison with commercial ELISA kits was performed using a subset of chicken and dog serum samples, and the paired results are summarized in Tables 2 and 3. Using the commercial ELISA as the reference comparator, the rSAG2–GICA showed a diagnostic sensitivity of 100.0% (3/3), diagnostic specificity of 90.7% (39/43), and overall agreement of 91.3% (42/46; 95% CI: 79.2%–97.6%) for chicken sera (Table 2; *n* = 46) when compared with the Chicken Tox‐Ab ELISA Kit. Notably, all ELISA‐positive chicken sera were also identified as positive by rSAG2–GICA, whereas four samples were positive by rSAG2–GICA but negative by ELISA. For dog sera (Table 3; *n* = 44), the assay showed a diagnostic sensitivity of 100.0% (1/1), diagnostic specificity of 97.7% (42/43), and overall agreement of 97.7% (43/44; 95% CI: 88.0%–99.9%) when compared with the Canine Tox‐Ab ELISA Kit. Similarly, the single ELISA‐positive dog serum sample was also positive by rSAG2–GICA, while one additional sample was positive by rSAG2–GICA but negative by ELISA. McNemar’s exact test showed no significant difference between the paired results in either chicken or dog sera (*p* = 0.125 and *p* = 1.000, respectively), indicating good preliminary concordance between rSAG2–GICA and the corresponding commercial ELISA kits.

**Table 2:** Agreement between rSAG2–GICA and a commercial ELISA kit for detection of *T. gondii* antibodies in chicken sera.

Method and results		Chicken Tox‐Ab ELISA		
		Positive	Negative	Total
rSAG2–GICA	Positive	3	4	7
	Negative	0	39	39
	Total	3	43	46
	Diagnostic sensitivity (%)	100	—	—
	Diagnostic specificity (%)	—	90.7	—
	Overall agreement (%)	—	—	91.3

*Note:* Diagnostic sensitivity, diagnostic specificity, and overall agreement were calculated using the commercial ELISA as the reference comparator. Diagnostic sensitivity was calculated as the proportion of ELISA‐positive samples that were also positive by rSAG2–GICA, whereas diagnostic specificity was calculated as the proportion of ELISA‐negative samples that were also negative by rSAG2–GICA. Overall agreement was calculated as the proportion of samples with concordant results between rSAG2–GICA and commercial ELISA among all tested samples. The 95% confidence interval (95% CI) for overall agreement was calculated using the exact Clopper–Pearson binomial method. Paired comparison between rSAG2–GICA and commercial ELISA was performed using McNemar’s exact test.

**Table 3:** Agreement between rSAG2–GICA and a commercial ELISA kit for detection of *T. gondii* antibodies in dog sera.

Method and results		Canine Tox‐Ab ELISA		
		Positive	Negative	Total
rSAG2–GICA	Positive	1	1	2
	Negative	0	42	42
	Total	1	43	44
	Diagnostic sensitivity (%)	100	—	—
	Diagnostic specificity (%)	—	97.7	—
	Overall agreement (%)	—	—	97.7

*Note:* Diagnostic sensitivity, diagnostic specificity, and overall agreement were calculated using the commercial ELISA as the reference comparator. Diagnostic sensitivity was calculated as the proportion of ELISA‐positive samples that were also positive by rSAG2–GICA, whereas diagnostic specificity was calculated as the proportion of ELISA‐negative samples that were also negative by rSAG2–GICA. Overall agreement was calculated as the proportion of samples with concordant results between rSAG2–GICA and commercial ELISA among all tested samples. The 95% confidence interval (95% CI) for overall agreement was calculated using the exact Clopper–Pearson binomial method. Paired comparison between rSAG2–GICA and commercial ELISA was performed using McNemar’s exact test.

## 4. Discussion

Toxoplasmosis is a globally distributed zoonotic infection caused by *T. gondii* and remains an important One Health concern. The disease inflicts severe economic losses on the global livestock industry, estimated at approximately $3 billion annually from abortion alone, while also threatening the health and welfare of companion animals [20, 21]. Importantly, both livestock/poultry and companion animals contribute to human exposure risk through the consumption of undercooked meat containing tissue cysts and through environmental contamination with oocysts shed by felids. Consequently, effective monitoring of infection in animal populations is essential for risk assessment, timely intervention, and the prevention and control of human toxoplasmosis.

Despite this need, effective monitoring is often constrained by the limited availability of rapid, field‐deployable, and multispecies diagnostic tools. Although ELISA and PCR provide reliable performance, they typically require specialized equipment, trained personnel, and relatively long turnaround times, limiting their suitability for on‐site screening and large‐scale surveillance [22]. Moreover, conventional serological assays are frequently species‐restricted because they rely on host‐specific secondary antibodies; thus, multispecies testing often requires multiple species‐specific kits, which is inconvenient and costly [23]. To address these limitations, we developed an rSAG2‐based double‐antigen sandwich colloidal GICA that enables rapid, visual, and species‐independent detection without secondary antibodies. In this format, rSAG2 serves as both the capture antigen and the gold‐labeled probe, allowing antibody detection via universal antigen‐antibody binding. The assay was validated using clinical sera from six animal species (chickens, pigs, sheep, yaks, dogs, and cats) and demonstrated favorable analytical performance and storage stability; future work will extend validation to additional hosts, including humans, to further support integrated One Health surveillance.

The performance of serological assays is highly dependent on the antigen selection. Although *T. gondii* is a single species, it comprises three major clonal genotypes (I, II, and III); therefore, the use of a genotype‐conserved antigen is critical to ensure broad and reliable detection across circulating strains. In this study, we selected SAG2 as the diagnostic target. SAG2 is a surface‐associated protein involved in host cell invasion and has been widely recognized as an immunodominant antigen [24]. Importantly, SAG2 contains conserved epitopes across different genotypes, enabling antibody recognition in infections caused by diverse *T. gondii* lineages. In addition, SAG2 is expressed during the early stages of infection, which supports sensitive serological detection aligned with the development of the humoral response. Its functional relevance is further supported by the evidence that anti‐SAG2 antibodies can inhibit parasite invasion [25]. Based on these attributes, we produced high‐purity rSAG2 by prokaryotic expression and used it as the core reagent to establish the rSAG2‐based double‐antigen sandwich GICA. This antigen‐choice strategy provides technical support for simultaneous detection of antibodies elicited by multiple major *T. gondii* genotypes and strengthens the applicability of the assay for broad‐spectrum surveillance.

In this study, we applied the assay to clinical sera from six animal species (chickens, dogs, cats, pigs, yaks, and sheep) and further benchmarked its performance against commercial ELISA kits in subsets of chicken and dog sera. The high overall agreement with ELISA (91.3% for chicken sera and 97.7% for dog sera) supports preliminary diagnostic consistency with routine laboratory serology. Notably, all ELISA‐positive samples in both subsets were also identified as positive by rSAG2‐GICA, whereas a small number of discordant samples were GICA‐positive but ELISA‐negative. Together with the nonsignificant McNemar’s exact test results, these findings indicate good preliminary agreement between the two methods. In addition, the strip’s instrument‐free workflow and visual readout within 15 min provide clear operational advantages for primary veterinary clinics, farms, and field epidemiological investigations where laboratory infrastructure is limited [26].

Among the tested food‐producing animals, relatively high seropositivity was observed in yaks (31.3%), sheep (26.0%), and pigs (25.9%). Although host species, breeding system, and sampling region were not independently evaluated in the present study, these patterns may reflect differences in management practices and opportunities for environmental exposure to oocysts. From a public health perspective, the persistence of *T. gondii* in food animals may increase the probability of human exposure through the food chain, particularly where undercooked meat consumption occurs [27–30]. Notably, the seroprevalence in pigs was lower than some previously reported national estimates, which may be related to improved biosecurity and management associated with intensive production systems [31, 32]. In this context, a rapid field test can facilitate routine monitoring, identification of higher‐risk production systems, and evaluation of control measures aimed at reducing both animal and human risks.

In poultry, the overall chicken seroprevalence (7.0%) was lower than some previously reported estimates, and seropositivity was descriptively higher in free‐range chickens than in intensively reared chickens, supporting the view that housing conditions and ground‐foraging behavior may influence exposure to oocysts [33, 34]. Because poultry production often includes smallholder or free‐range practices where environmental control is challenging, rapid on‐site screening may be particularly valuable for identifying high‐risk flocks and informing practical biosecurity measures that reduce exposure and potential spillover risk.

In companion animals, seropositivity was relatively low (2.9% in dogs and 3.3% in cats), which may be related to more limited environmental exposure in the sampled population. Nonetheless, companion animals remain important from a public health standpoint, particularly cats as definitive hosts responsible for oocyst shedding; therefore, surveillance in dogs and cats can provide actionable information about household‐ and community‐level exposure risk and can support targeted risk communication to pet owners. In such settings, a rapid and accessible test may help broaden surveillance coverage beyond specialized laboratories.

However, several limitations of the present study should be acknowledged. First, comparator‐based evaluation was performed only in subsets of sera from two animal species and was not extended to all host groups included in this study. Second, the number of ELISA‐positive samples available for comparison was limited, particularly in the chicken and dog subsets, which reduced the robustness of the diagnostic sensitivity and agreement estimates. Third, the positive sera used for assay development and detection‐limit evaluation were characterized by indirect ELISA but were not further confirmed by another established serological method such as MAT. In addition, standardized positive reference sera were not available; therefore, the detection‐limit result based on *T. gondii*‐infected rat serum should be interpreted as a preliminary and serum‐dependent estimate. Fourth, the analytical specificity assessment was based on the tested serum panel and therefore does not exclude the possibility of cross‐reactivity with other untested conditions. In addition, the current assay relied on visual interpretation of strip results, which may introduce a certain degree of subjectivity, especially for weakly positive samples. Therefore, further validation using larger and more diverse sample sets, broader host categories, and more standardized reference methods will be necessary to more comprehensively assess the diagnostic performance and field applicability of this assay.

## Author Contributions

Xin Mu carried out major experiments, data collection/analysis, and initial writing. Chen Chen edited the manuscript and supported experiments. Xianglin Pu managed cell culture and *T. gondii* maintenance. Lixin Xu, Mingmin Lu, Ruofeng Yan, and Xiangrui Li offered strategic and technical guidance. Xiaokai Song provided overall supervision, design approval, funding coordination, and manuscript approval.

## Funding

This work was funded by the Jiangsu Provincial Science and Technology Plan Special Fund Key R&D Program—Social Development (Grant BE2023823).

## Ethics Statement

All animal care and usage procedures complied with the guidelines established by the Institutional Animal Care and Use Committee of Nanjing Agricultural University, under approval number NJAU. No. 20230322029.

## Conflicts of Interest

The authors declare no conflicts of interest.

## Supporting Information

Additional supporting information can be found online in the Supporting Information section.

## Supporting information


**Supporting Information 1** Table S1: Primers for SAG2 gene amplification. Primers used for amplification of the SAG2 gene, including nucleotide sequences, restriction enzyme sites, and amplicon size.

**Supporting Information 2** Figure S1: PCR amplification of SAG2, restriction analysis of the recombinant plasmid, and distribution of recombinant SAG2 protein. (A) Agarose gel electrophoresis showing the PCR product of the SAG2 gene and diagnostic double‐enzyme digestion of the recombinant plasmid. M1, DL 2500 DNA marker; lane 1, PCR‐amplified SAG2 fragment (arrow); M2, DL 5000 DNA marker; lane 2, double‐digested recombinant plasmid yielding bands corresponding to the insert and vector backbone (arrows). (B) SDS–PAGE analysis of recombinant SAG2 expression and its distribution in different fractions. Lane 1, soluble fraction (supernatant) after cell lysis; lane 2, insoluble fraction (pellet/inclusion bodies).

**Supporting Information 3** Figure S2: Optimization of labeling pH and antigen amount. (A) Determination of optimal labeling pH. Representative photographs show the color of colloidal gold solutions at different pH values (tubes 1‐10 correspond to pH 3.5, 4.0, 4.5, 5.0, 5.5, 6.0, 6.5, 7.0, 7.5, and 8.0). The line plot shows the corresponding OD_520_ values; the optimal labeling pH was defined as the lowest pH that prevented salt‐induced aggregation (final NaCl concentration, 5%) and yielded the highest OD_520_. (B) Determination of optimal antigen labeling amount. Representative photographs show the color of colloidal gold solutions incubated with increasing rSAG2 concentrations (tubes 1‐10 correspond to 0, 6.4, 12.8, 19.2, 25.6, 32.0, 38.4, 44.8, 51.2, and 57.6 μg/mL). The line plot shows the corresponding OD_520_ values; the optimal labeling amount was defined as the minimum rSAG2 concentration that prevented salt‐induced aggregation and produced the highest OD_520_.

**Supporting Information 4** Figure S3: Specificity of the rSAG2‐GICA strips against 23 common animal pathogens. Representative photographs show the results of the rSAG2‐GICA strips tested with *T. gondii*‐positive serum (positive control), negative serum (negative control), and sera positive for 23 common animal pathogens. In the upper row, the strips were tested with sera positive for *Eimeria tenella, E. necatrix, E. maxima, E. acervulina*, infectious bursal disease virus (IBDV), Newcastle disease virus (NDV), *Salmonella* spp., *Pasteurella multocida, Trichinella spiralis, Haemonchus contortus*, and *Actinobacillus pleuropneumoniae*. In the lower row, the strips were tested with sera positive for classical swine fever virus (CSFV), pseudorabies virus (PRV), porcine reproductive and respiratory syndrome virus (PRRSV), porcine circovirus (PCV), Streptococcus suis, Glaesserella parasuis, canine parvovirus (CPV), canine adenovirus (CADV), canine influenza virus (CIV), feline calicivirus (FCV), feline parvovirus (FPV), and feline herpesvirus (FHV). The experiment was performed in duplicate.

## Data Availability

The data that support the findings of this study are available from the corresponding author upon reasonable request.
