# Supplementary material for: Development and Field Validation of a Double‐Antigen Sandwich Colloidal Gold Immunochromatographic Strip for Detection of Toxoplasma gondii Antibodies in Multiple Host Species
Source: Transbound Emerg Dis. 2026 Jun 2;2026:5879710. doi: 10.1155/tbed/5879710 (PMC13239236; doi:10.1155/tbed/5879710)
Supplement: Supplementary file 1 — Supporting Information 1 Table S1: Primers for SAG2 gene amplification. Primers used for amplification of the SAG2 gene, including nucleotide sequences, restriction enzyme sites, and amplicon size. [file TBED-2026-5879710-s003.docx]

Table S1 Primers for SAG2 gene amplification

| Primer | Primer sequence | Restriction enzyme | Target fragment/bp |
| --- | --- | --- | --- |
| Forward | 5’-CGAGCTCTCCACCACCGAGACG-3’ | *Sac* I | 480 |
| Reverse | 5’-CCCAAGCTTCGTGAGAGACACAGGGTC-3’ | *Hin*d III |  |
