# Supplementary figures and images for: Development and Field Validation of a Double‐Antigen Sandwich Colloidal Gold Immunochromatographic Strip for Detection of Toxoplasma gondii Antibodies in Multiple Host Species
Source: Transbound Emerg Dis. 2026 Jun 2;2026:5879710. doi: 10.1155/tbed/5879710 (PMC13239236; doi:10.1155/tbed/5879710)

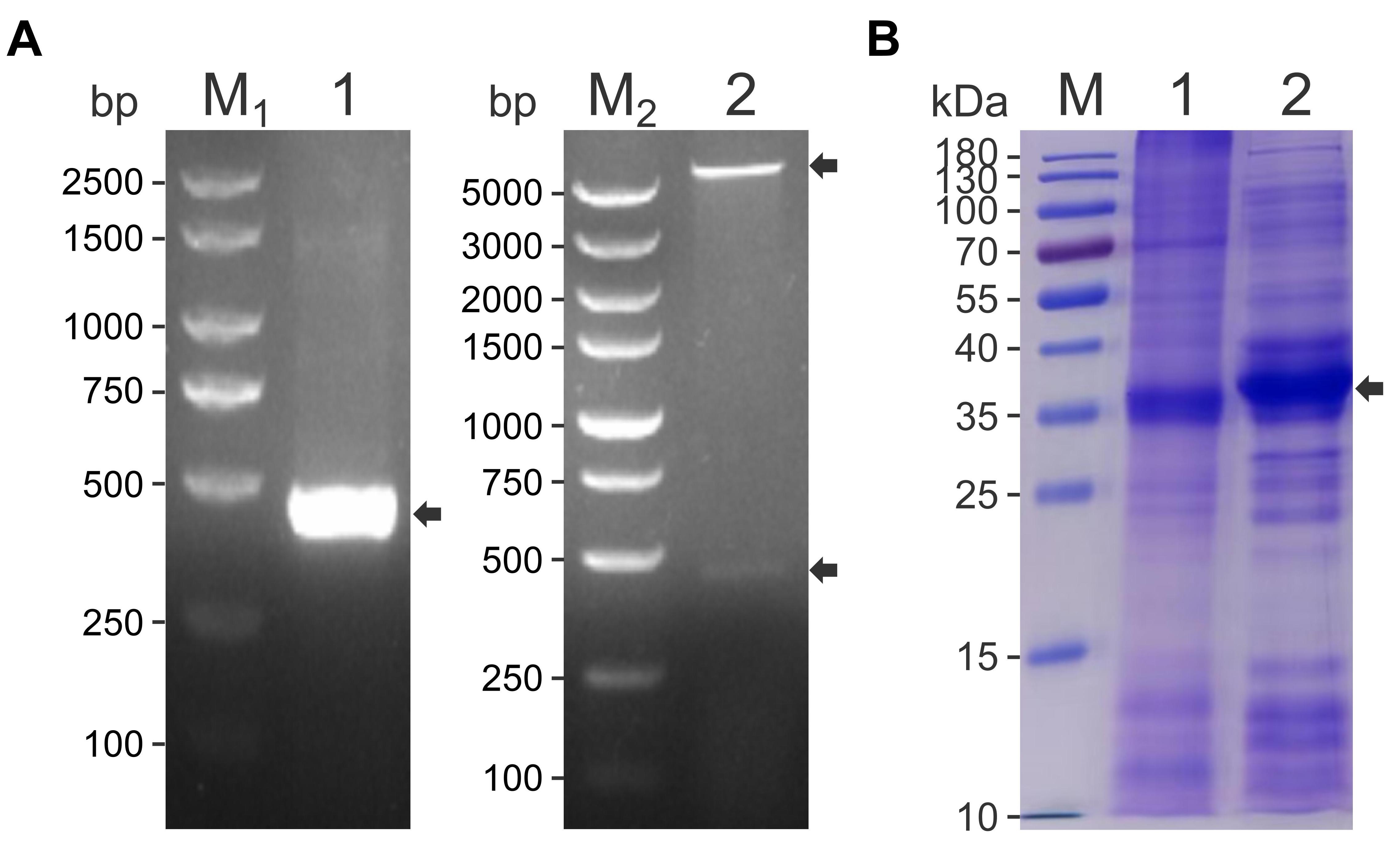

Supplement: Supplementary file 2 — Supporting Information 2 Figure S1: PCR amplification of SAG2, restriction analysis of the recombinant plasmid, and distribution of recombinant SAG2 protein. (A) Agarose gel electrophoresis showing the PCR product of the SAG2 gene and diagnostic double‐enzyme digestion of the recombinant plasmid. M1, DL 2500 DNA marker; lane 1, PCR‐amplified SAG2 fragment (arrow); M2, DL 5000 DNA marker; lane 2, double‐digested recombinant plasmid yielding bands corresponding to the insert and vector backbone (arrows). (B) SDS–PAGE analysis of recombinant SAG2 expression and its distribution in different fractions. Lane 1, soluble fraction (supernatant) after cell lysis; lane 2, insoluble fraction (pellet/inclusion bodies). [file TBED-2026-5879710-s002.jpg]

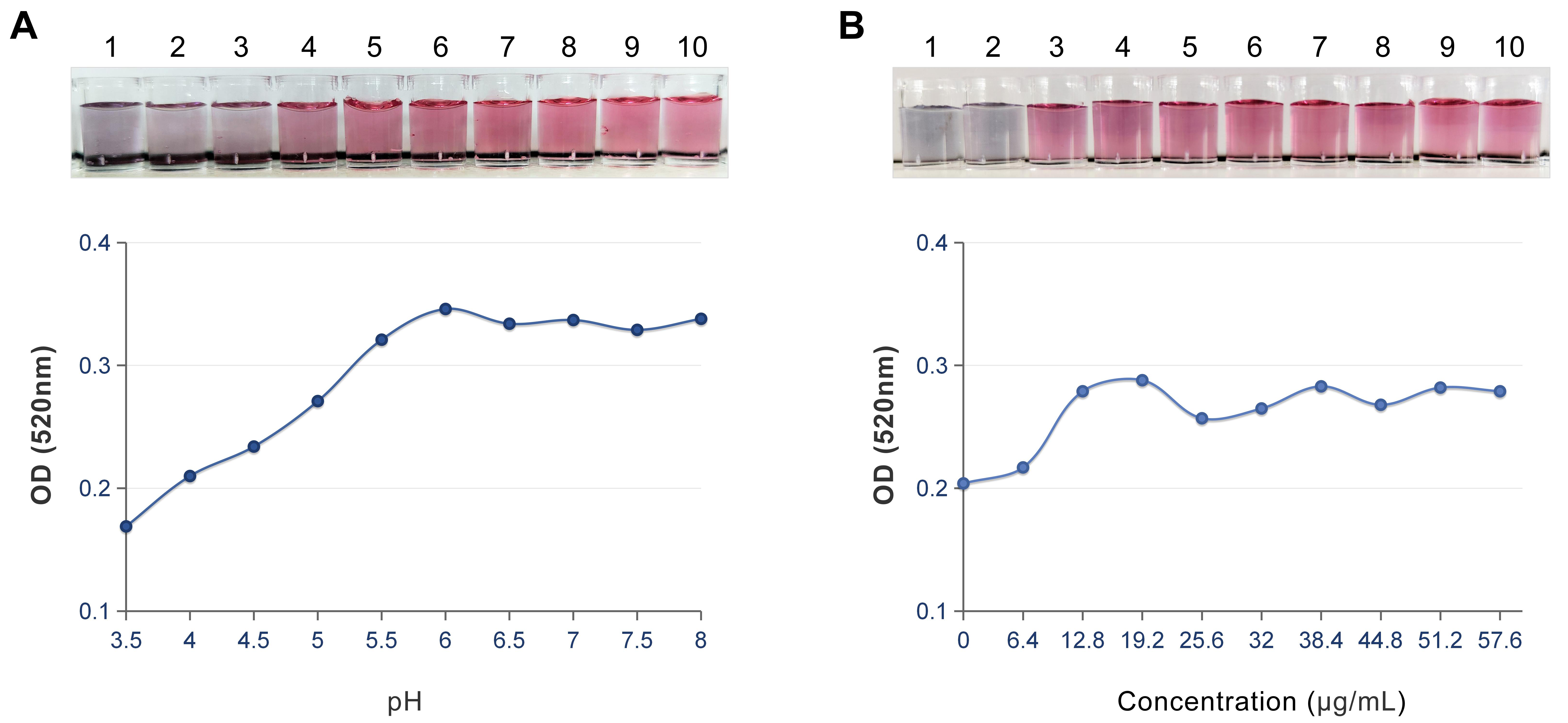

Supplement: Supplementary file 3 — Supporting Information 3 Figure S2: Optimization of labeling pH and antigen amount. (A) Determination of optimal labeling pH. Representative photographs show the color of colloidal gold solutions at different pH values (tubes 1‐10 correspond to pH 3.5, 4.0, 4.5, 5.0, 5.5, 6.0, 6.5, 7.0, 7.5, and 8.0). The line plot shows the corresponding OD520 values; the optimal labeling pH was defined as the lowest pH that prevented salt‐induced aggregation (final NaCl concentration, 5%) and yielded the highest OD520. (B) Determination of optimal antigen labeling amount. Representative photographs show the color of colloidal gold solutions incubated with increasing rSAG2 concentrations (tubes 1‐10 correspond to 0, 6.4, 12.8, 19.2, 25.6, 32.0, 38.4, 44.8, 51.2, and 57.6 μg/mL). The line plot shows the corresponding OD520 values; the optimal labeling amount was defined as the minimum rSAG2 concentration that prevented salt‐induced aggregation and produced the highest OD520. [file TBED-2026-5879710-s001.jpg]

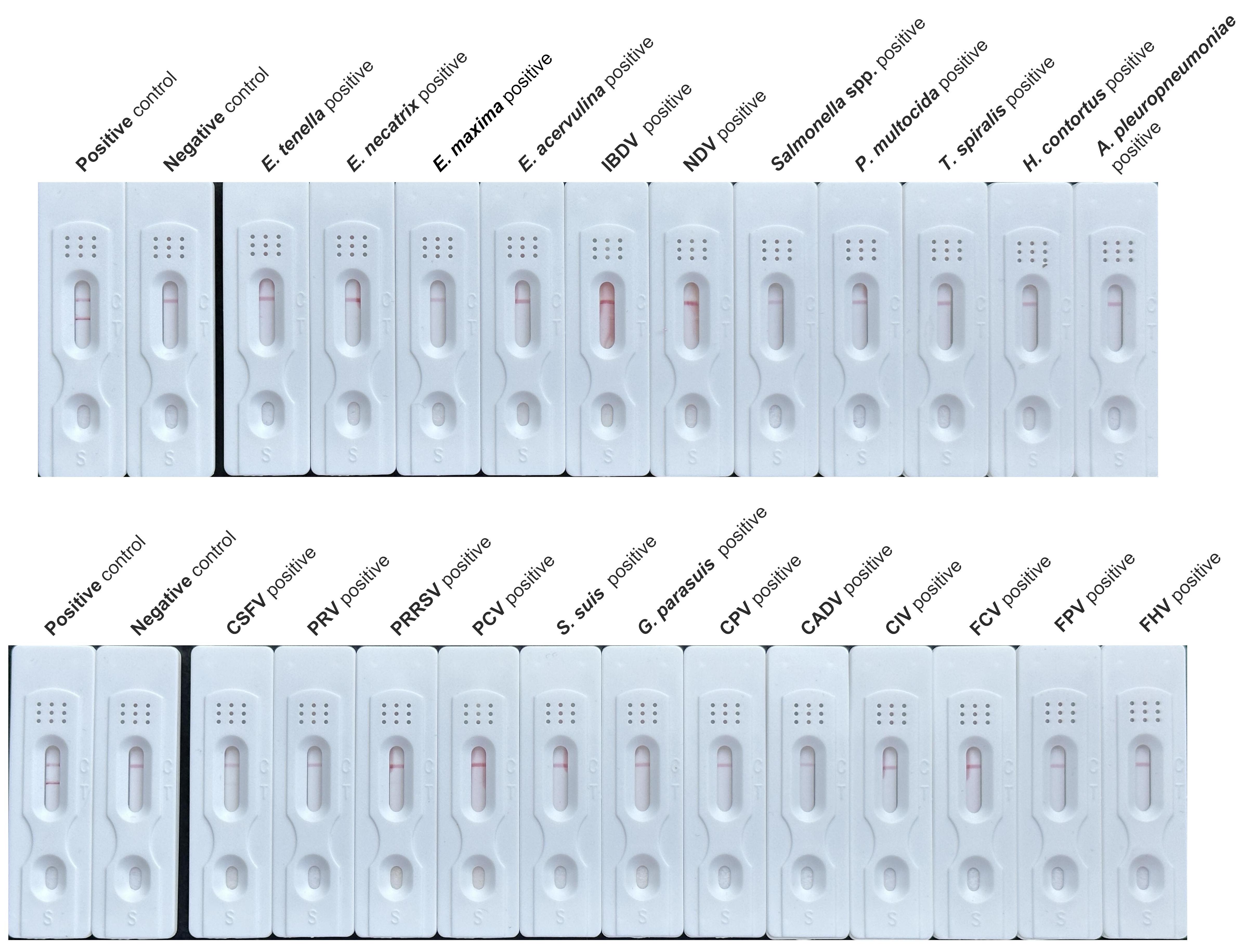

Supplement: Supplementary file 4 — Supporting Information 4 Figure S3: Specificity of the rSAG2‐GICA strips against 23 common animal pathogens. Representative photographs show the results of the rSAG2‐GICA strips tested with T. gondii‐positive serum (positive control), negative serum (negative control), and sera positive for 23 common animal pathogens. In the upper row, the strips were tested with sera positive for Eimeria tenella, E. necatrix, E. maxima, E. acervulina, infectious bursal disease virus (IBDV), Newcastle disease virus (NDV), Salmonella spp., Pasteurella multocida, Trichinella spiralis, Haemonchus contortus, and Actinobacillus pleuropneumoniae. In the lower row, the strips were tested with sera positive for classical swine fever virus (CSFV), pseudorabies virus (PRV), porcine reproductive and respiratory syndrome virus (PRRSV), porcine circovirus (PCV), Streptococcus suis, Glaesserella parasuis, canine parvovirus (CPV), canine adenovirus (CADV), canine influenza virus (CIV), feline calicivirus (FCV), feline parvovirus (FPV), and feline herpesvirus (FHV). The experiment was performed in duplicate. [file TBED-2026-5879710-s004.jpg]
